# Minimally Guided App-Based Intervention for Nontreatment-Seeking Psychologically Distressed Adults: Protocol for a Randomized Controlled Trial

**DOI:** 10.2196/85267

**Published:** 2026-06-05

**Authors:** Paulomi Sudhir, Abhishek Karishiddimath, Pramita Sengupta, Angelina Francis, Sravya Buddhavarapu, Jagadisha Thirthalli, Srikanth TK, Rajesh Sagar, Seema Mehrotra

**Affiliations:** 1 Department of Clinical Psychology National Institute of Mental Health and Neurosciences Bengaluru India; 2 Department of Psychiatry National Institute of Mental Health and Neurosciences Bengaluru India; 3 E-Health Research Centre International Institute of Information Technology Bangalore Bengaluru India; 4 Department of Psychiatry All India Institute of Medical Sciences Delhi India

**Keywords:** mental health, digital interventions, self-help app, randomized controlled trial, help-seeking behavior

## Abstract

**Background:**

Mental health conditions impose a significant burden worldwide, often remaining untreated due to multiple demand- and supply-side barriers such as stigma, limited awareness, and poor access to services. Mobile-based interventions offer a scalable solution to address some of these barriers, including low rates of help-seeking. However, most evidence has emerged from high-income countries, with minimal research in the Indian setting, particularly among psychologically distressed individuals not currently seeking treatment.

**Objective:**

This study aims to evaluate the effectiveness of a minimally guided mobile app in promoting help-seeking behaviors among individuals experiencing self-reported common mental health concerns and significant psychological distress who are not currently engaged in formal treatment. Additionally, it will examine the utility of the intervention in reducing perceived barriers to seeking professional help for mental health, improving the inclination to seek help from mental health professionals, and alleviating psychological distress. Perceived benefits of using the app will also be documented.

**Methods:**

A parallel-arm randomized controlled trial will be used to evaluate the effectiveness of the minimally guided, indigenously developed mobile app in enhancing help-seeking behaviors among distressed, nontreatment-seeking adults. The intervention arm will receive a multimodule app while the active control will receive a mood-logging app. Assessments will occur at baseline, 5 weeks after the intervention, and at the 1-month follow-up using standardized self-report tools and additional items. The primary outcome will be actual help-seeking behavior in accessing mental health professionals, while secondary outcomes will include help-seeking inclination, perceived barriers, psychological distress, and perceived benefits.

**Results:**

The trial commenced in September 2025 and is currently in the recruitment and data collection phase. Participant enrollment is ongoing. Data collection is anticipated to be completed by July 2026, and findings from the study are expected to be disseminated through a peer-reviewed publication by December 2026.

**Conclusions:**

This study will provide contextually relevant insights into the utility of a minimally guided mobile app-based intervention in improving professional help-seeking among psychologically distressed, nontreatment-seeking individuals in India. Findings may inform the design and scaling of accessible digital interventions to bridge the mental health treatment gap in low-resource settings.

**Trial Registration:**

Clinical Trial Registry of India CTRI/2024/11/077461; https://tinyurl.com/2edf5a32

**International Registered Report Identifier (IRRID):**

DERR1-10.2196/85267

## Introduction

### Background

Despite the availability of effective treatments, common mental health problems such as depression remain highly prevalent, with a significant treatment gap persisting both globally and in India [1,2]. This has been attributed to both supply-side and demand-side barriers, such as poor access to mental health services, low mental health literacy, and stigma [3]. Noting that global efforts have disproportionately focused on specialist psychiatric care, the World Health Organization model of the optimal mix of services highlights the importance of investing efforts in the development of low-intensity interventions such as self-help and community support for milder severity of problems [4]. In addition to strengthening access to mental health services, research highlights the need for scalable interventions that address demand-side barriers, including efforts to improve mental health literacy and encourage help-seeking [2-4]. In the context of mental health, help-seeking has been defined as “an adaptive coping process—that is, the attempt to obtain external assistance (from informal and formal sources of help) to deal with a mental health concern” [5]. A variety of interventions have been developed to improve mental health help-seeking, often targeting either an individual’s inclination (the willingness to seek help) or behavior (the actual act of seeking care) [6].

Digital mental health interventions have emerged as promising avenues for delivering low-intensity self-help strategies, enhancing mental health literacy, and improving help-seeking due to advantages such as scale, privacy, and ease of access [7]. There is burgeoning research on app-supported interventions for various mental health problems across diverse populations, suggesting their utility as scalable and accessible tools to address the unmet mental health needs of populations [8-11]. However, most research on digital interventions originates from high-income countries, with a notable scarcity of studies from low- and middle-income regions [12-14].

There is a scarcity of studies from the Indian context on the utility of mobile app-based interventions for common mental health concerns, and particularly for enhancing help-seeking [15-17].

A recent systematic review of mental health apps accessible to Indian users through major app stores examined 350 apps in detail [18]. Out of these, the largest numbers were contributed by apps retrieved using the keywords “depression,” “anxiety,” and “mental health.” Only 17.7% of the reviewed apps originated from Asia, underscoring the limited regional representation in digital tools available to Indian users. This review highlighted several critical concerns. Notably, 65% of apps did not mention any involvement of mental health professionals in their development. Only 11% referenced an evidence base, and issues related to privacy and user safety were also prominent. Although about 40% of apps included pointers to seek professional help, these were often limited to generic disclaimers. Proactive nudges or motivational prompts to seek help appeared in fewer than a quarter of the reviewed apps. These observations point to critical gaps in the development of mental health mobile apps for Indian users.

Against this background, an indigenous mobile self-help app, called MindNotes, was developed for the Indian context, informed by prior research and rooted in the clinical experience of mental health professionals working in the country, and based on formative work [19]. The app evolved through multiple iterations and user feedback. It is among the first indigenous apps available in English, as well as 2 Indian languages (Kannada and Hindi), thus aiming to address linguistic diversity in the country to some extent. It is designed for individuals experiencing psychological distress or common mental health symptoms who may be uncertain about the nature or severity of their concerns, or ambivalent about seeking professional support. It is a multimodule app containing sections aimed at (1) improving self-awareness about the nature and severity of common mental health concerns and receiving recommendations on the need for professional help (self-discovery section), (2) identifying and reducing barriers to seeking professional help (breaking barriers section), and (3) improving knowledge and skills in using self-help strategies for common mental health concerns, such as managing worry and self-criticality (self-help toolkit). Additional sections include the coping with the crisis section, which provides simple strategies for dealing with a crisis and accessing helpline numbers, and the professional connect section, which clarifies general doubts and queries regarding mental health and the process of professional consultation, offers basic support, and provides nudges to seek professional help via a note-sharing space.

The app involves interactive elements and includes quizzes, personalized feedback, motivational videos, reflective exercises, self-help strategies, and guidance related to common mental health concerns. A preliminary trial of this app indicated encouraging trends in the perceived relevance and potential usefulness of its various sections and their utility in improving mental health awareness and motivation to seek professional consultation for self-reported depressive and anxiety symptoms [20].

Unguided digital interventions tend to result in low engagement rates that can influence outcomes [21,22]. The MindNotes app provides minimal guidance and support through its professional connect module for users. In the experience of the authors, the content shared by users on the professional connect section underscores its value in reaching distressed, nontreatment-seeking individuals seeking information, support, and guidance. Its self-discovery and breaking barriers sections are aimed at helping users gain a better understanding of their depressive or anxiety symptoms and overcome hesitations and barriers to seeking professional help when required. Thus, the app has the potential to strengthen help-seeking inclinations and behaviors, while also offering self-help strategies for common mental health concerns that may contribute to reductions in distress. Moreover, it is well recognized that interventions to improve help-seeking are likely to be useful when these are delivered as targeted interventions for those experiencing symptoms or significant distress [23].

This study is aimed at examining the effectiveness of the MindNotes app in improving help-seeking in a sample of distressed nontreatment seekers by using a randomized controlled design.

### Objectives

The objectives of the study are to evaluate the effectiveness of the minimally guided MindNotes app in enhancing professional help-seeking behaviors among individuals with self-reported common mental health concerns and significant psychological distress who are not currently engaged in treatment. Additionally, the study aims to examine the utility of this app in improving the inclination to seek help from mental health professionals, reducing perceived barriers to seeking professional help for mental health, reducing psychological distress, and documenting perceived benefits of using the app. Help-seeking behavior is the primary outcome variable, while help-seeking inclination, barriers to seeking professional help, and psychological distress are the secondary outcome variables.

## Methods

### Study Design

This study will use a parallel-arm randomized controlled design with assessments at baseline, after the intervention, and at follow-up to compare the effectiveness of the minimally guided MindNotes app-based intervention against an active control condition involving a basic mood-logging intervention described further. The trial will be conducted over a period of 4 weeks, with reminders provided at specific intervals to support participant engagement in both the arms. Participants will complete the postintervention assessment at the conclusion of the 4‑week intervention phase, with a follow-up assessment scheduled 1 month after the postintervention assessment. The study design and reporting will adhere to the SPIRIT (Standard Protocol Items: Recommendations for Intervention Trials) guidelines for randomized trials of digital health interventions [24].

### Recruitment and Sampling

Participants will be recruited from the community through a multipronged strategy combining online and offline dissemination. This will include social media outreach, online announcements, and dissemination of posters in colleges, workplaces, and community locations. The inclusion criteria for the study are individuals aged 18 years and older, of all genders, fluent in English or Kannada, with at least 10 years of formal education, comfortable using a smartphone, experiencing self-reported current depressive or anxiety symptoms for 2 weeks or longer, and scoring 20 or above on the Kessler Psychological Distress Scale (K10) [25]. Participants will be excluded if they are currently receiving pharmacological treatment for any mental health condition, have attended 3 or more psychotherapy or counseling sessions in the past 3 months, or are presently engaged in counseling or psychotherapy. Individuals currently using any other mental health app, or those not amenable to participating in the study as assessed in a brief telephonic interview, or those having significant suicidal ideation as determined by responses on 2 items pertaining to the frequency and controllability of suicidal thoughts in the past 1 month, will be excluded from the study.

### Power Calculation

A sample size of 150 participants per group has been estimated based on detecting a difference in help-seeking behavior (25% vs 10%) with 80% power at a 5% significance level using G*Power 3.1, allowing for up to 33% attrition commonly observed in minimally guided digital interventions [26]. These include interventions delivered through digital platforms, which often vary widely in design and outcomes. Consequently, closely comparable studies for precise sample size estimation were difficult to identify, and the current estimate is informed by findings from studies with broadly similar characteristics [14,27].

### Procedure

Participants will be randomized into either the intervention or control group in a 1:1 ratio, using a computer-generated randomization sequence. The randomization list will be generated by the study supervisor. Stratified randomization will be used based on age group (18-35 years and 36 years and above) and gender to ensure balance across key demographic variables.

A brief telephonic interview for eligibility screening will be conducted after all other questionnaires are completed. This call allows the researchers to clinically assess the current mental state and amenability to take part in the study process, inquire whether participants have sought consultation for any psychiatric problem in the past or recent times, and confirm willingness to participate.

**Figure 1:**
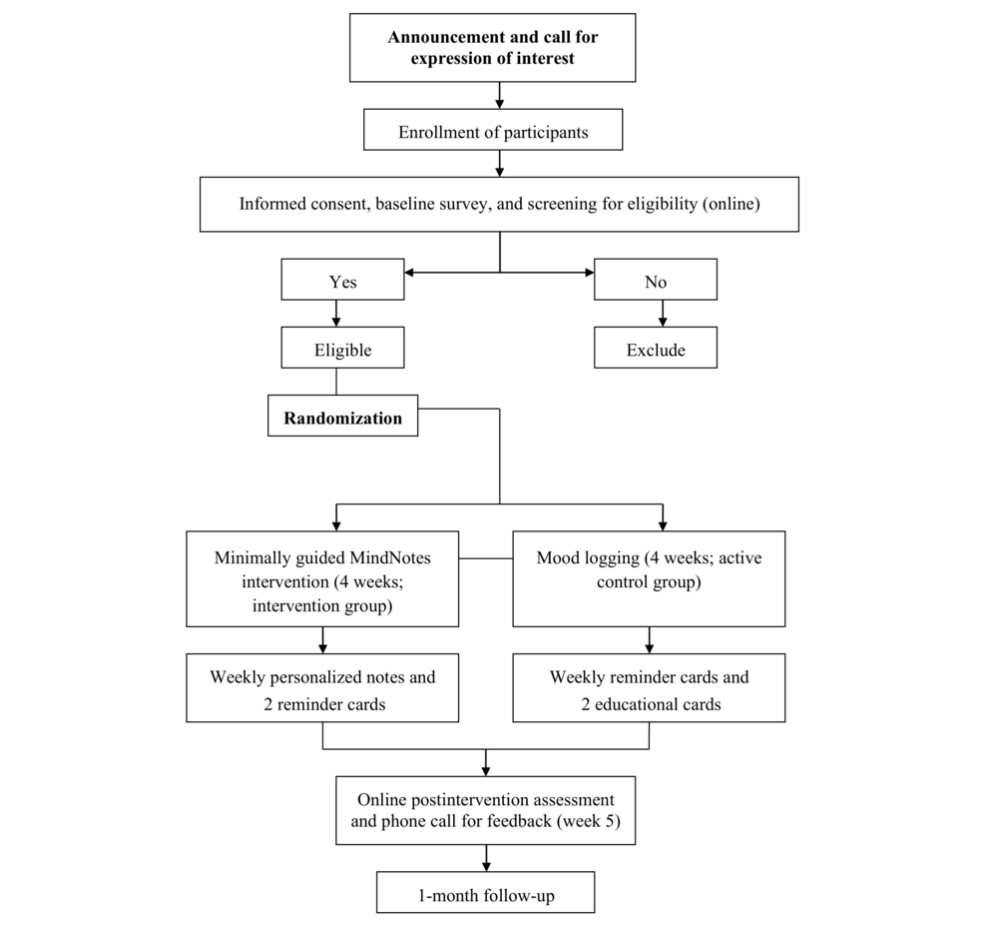
Flowchart of the study procedure.

Eligible participants will receive an introductory call informing them of their group allocation and guiding them on how to begin engagement with the study activities. All assessments will be conducted through an online survey, and feedback will be obtained over the phone from both groups at the end of the 4-week intervention period. A follow-up telephonic interview will be conducted 1 month after the postintervention assessment to ascertain help-seeking inclination and help-seeking behavior. The flow of the study is depicted in Figure 1.

### Interventions

#### Overview of MindNotes (Minimally Guided App-Based Intervention)

MindNotes has been described in an earlier section. It is a multimodule mobile app with sections designed to enhance self-awareness of mental health concerns, reduce help-seeking barriers, and build self-help skills. The intervention group will be requested to complete the self-discovery section of the MindNotes app and explore the breaking barriers section, as well as any other self-help sections as per their preference over 4 weeks. During this period, 2 reminders to encourage engagement with the app will be sent over WhatsApp (Meta Platforms). As part of the minimal guidance strategy, 4 brief personalized notes will be sent from the professional connect section of the app on a once-a-week basis to educate, clarify doubts, and motivate app use. The professional connect messages will be personalized based on the participant’s baseline distress, self-reported concerns, and their exploration of various sections of the MindNotes app. These messages will involve the communication of empathy, prompts to explore various sections of the app, and encouragement to apply what is learned through app exploration.

#### Overview of Mood Monitoring–Based Intervention

Mood monitoring apps can serve as credible active control conditions because they engage participants in regular self-reflection and emotional tracking without delivering targeted therapeutic content [28]. Moreover, mood tracking has been shown to improve emotional self-awareness and reduce depressive symptoms through increased insight and regulation, making it a psychologically active but nonspecific comparator in this study context involving help-seeking behavior as the primary outcome variable [29].

The participants in the control group will be asked to engage with a mobile-responsive, web-based mood monitoring or mood-logging app for the same 4-week duration as the intervention group. They will be instructed to log their mood at least twice a day for 4 weeks on this mood monitoring app. This would involve rating their mood on a 7-point Likert scale measuring levels of pleasantness and unpleasantness and indicating the nature of their predominant emotion from a list of emotion words provided (eg, happy, guilty, angry, sad, etc). Additionally, they will receive 4 generic reminders to encourage mood tracking and engage in self-care, along with 2 educational messages focused on enhancing mental health awareness via WhatsApp. These messages will include basic and brief information on managing common mental health concerns without any personalized elements. Overall, the mood-logging web app, along with a few educational messages, is expected to serve as an active control by controlling for nonspecific factors such as user engagement, expectancy effects, and digital interaction while offering a credible low-intensity self-help strategy to gain self-awareness.

To summarize, in addition to the opportunity to engage with the respective apps (MindNotes or mood-logging web application), both groups will receive 6 messages through the 4-week period of the study. The intervention group will receive 2 generic reminders alongside 4 personalized professional connect messages. The control group will receive 4 generic reminders and 2 educational cards focused on mood tracking, self-awareness, and emotional well-being. These reminders and messages are strategically distributed throughout the 4-week study period to promote participant engagement with either the MindNotes app or the mood-logging app within a minimal guidance framework, as shown in Table 1.

**Table 1:** Details of the timing and contents of messages for both groups.

Day	Intervention group	Control group
6	Professional connect message 1: motivational message encouraging exploration of the “self-discovery” module and consistent app use	WhatsApp educational card 1: psychoeducation about mood tracking and encouragement to begin
10	WhatsApp reminder 1: inviting exploration of the “breaking barriers” section	WhatsApp reminder 1: promotes mood check-ins and reflection using the mood-tracking app
12	Professional connect message 2: reminder to continue exploring the app	WhatsApp reminder 2: encourages mood tracking and self-reflection at the 2-week mark
18	Professional connect message 3: based on the backend activity of each user, recommends sections to explore	WhatsApp educational card 2: offering general tips on managing moods
20	WhatsApp reminder 2: Promotes further engagement with self-help strategies	WhatsApp reminder 3: reiterating the importance of tracking mood and understanding emotional patterns
22	Professional connect message 4: final motivational message, encourages ongoing self-care and engaging in any remaining sections of the app	WhatsApp reminder 4: encourages end-of-trial reflection and sustaining self-monitoring practices

### Data Collection Instruments

The demographic and basic data sheet will be used to collect demographic information. In addition, the basic data sheet will include items assessing prior consultations with mental health professionals (if any), significant life events or ongoing stressors, and available sources of support during difficult times. To assess self-perceived common mental health concerns, participants will respond to an item regarding their current experience of depressive and/or anxiety symptoms. This item will inquire whether they are currently experiencing (1) persistent sadness or loss of interest in previously enjoyable activities; (2) frequent feelings of nervousness, tension, or anxiety; (3) both; or (4) none of the above. The duration of these concerns will also be captured using predefined categories ranging from less than 2 weeks to more than 6 months. Perceived severity of distress and its interference with daily functioning will be measured using a 5-point Likert-type scale, and another item will capture any current engagement in professional help-seeking. Participants will also respond to an open-ended item about the 3 self-help methods they use most frequently. Additional items rated on a 5-point Likert scale will assess the perceived usefulness of self-help strategies, self-rated perseverance in pursuing personal goals, and interest in learning self-help skills for mental health and well-being.

The K10 is a 10-item standardized measure of distress related to anxiety and depression symptoms over the past 4 weeks. It has demonstrated good psychometric properties in varied samples [25,30,31]. A total score of ≥20 indicates significant psychological distress and potential mental disorder [25]. The scores on this tool will be used to recruit eligible participants for the study (distressed nontreatment-seeking individuals). Participants who score 20 or above on K10 will be included in the study. The scores will also be used to document changes, if any, in psychological distress during the course of the study (secondary outcome).

Suicidal ideation—2 brief items developed for the study will be used to determine significant suicidality based on frequency and controllability. This is for screening out those who may need immediate support and referral. The first item asks, “In the past month, how often have you had thoughts about suicide?” with response options ranging from never (coded as 1), rarely or 1-2 times (coded as 2), sometimes (coded as 3), often (coded as 4), and all the time (coded as 5). The second item asks, “When feeling very sad or emotionally distressed, and thoughts of self-harm or suicide arise, how much control do you feel you have over acting on these thoughts?” Responses are rated as able to control easily (coded as 1), able to control with some difficulty (coded as 2), able to control with great difficulty (coded as 3), not able to control (coded as 4), and not applicable—do not experience such thoughts (coded as 5). All participants who report rarely having suicidal thoughts with great difficulty or no control; those who report sometimes having suicidal thoughts with great difficulty or no control, or those reporting often having suicidal thoughts, regardless of how much control they report, will be excluded.

Help-seeking inclinations will be assessed through the use of the General Help-Seeking Questionnaire (GHSQ). This 14-item scale will be used to assess the likelihood of seeking help from various sources (eg, parents, friends, helpline, and mental health professionals) for current mental health concerns or psychological distress, on a 7-point Likert scale [32]. Ratings on the item specific to mental health professionals will be the primary focus, although help-seeking inclination from various sources will also be documented. At follow-up, for the sake of minimizing respondent burden, help-seeking inclination only from mental health professionals will be assessed using the corresponding GHSQ items with a 7-point Likert-type scale.

The Barriers to Seek Professional Help for Mental Health Scale (BSPH-MHS) will be used to assess the severity and nature of perceived barriers to professional help-seeking for mental health concerns or psychological distress. It is a 28-item, 5-point Likert scale that evaluates perceived barriers across 4 domains: distress perception, stigma, service apprehensions, and instrumental obstacles with previous research supporting its reliability, construct, and convergent validity [30].

Help-seeking behavior from a mental health professional will be assessed at baseline, postintervention assessment, and follow-up through a single yes or no item. If help is sought, details on the nature of professional consultation, duration since first consultation, type of treatment, and satisfaction with consultation will be assessed [27]. If professional consultation for mental health concerns has not been sought since participation in the study, the reasons for not seeking consultation will be explored through an open-ended item.

### Feedback Items

Feedback items at the postintervention assessment will include ratings on the current severity of distress on a 5-point Likert-type scale, frequency of using the app in the previous 1 month (multiple-choice item), challenges in using the app regularly or frequently (open-ended item), ease of navigation, relevance, and usefulness of the content of the intervention (5-point Likert-type items). Perceived benefits of participating in the study will be assessed using a set of 10-point Likert-type items on improvements in knowledge about common mental health concerns and seeking professional consultation for mental health, reduction in hesitations to consult a mental health professional when needed, and improvement in motivation to consult a mental health professional relative to baseline. The likelihood of recommending the intervention to others who may be distressed but not seeking professional help will also be assessed using a 10-point Likert-type item. In addition, an open-ended question will be used to document the impact of participating in the intervention, if any.

**Figure 2:**
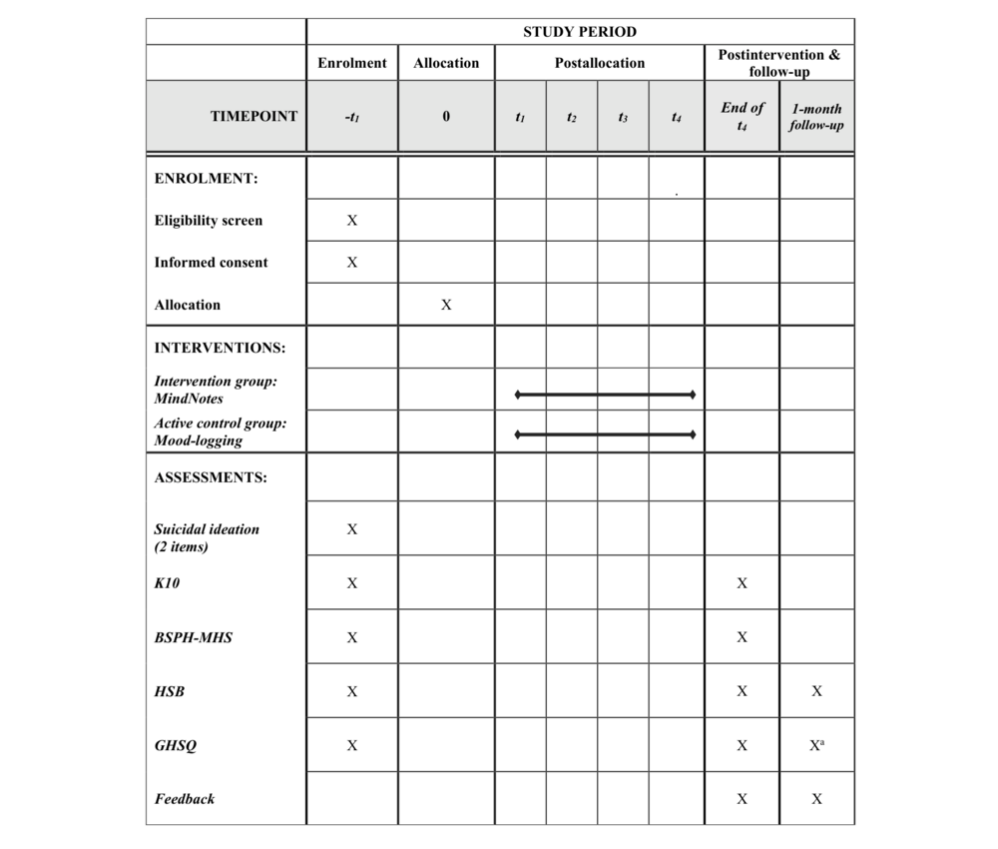
SPIRIT (Standard Protocol Items: Recommendations for Interventional Trials) diagram outlining the schedule of enrollment, intervention, and assessment. Suicidal ideation is assessed using 2 items developed by the researchers. The follow-up assessment includes only the General Help-Seeking Questionnaire (GHSQ) items assessing help-seeking inclination towards mental health professionals; this is indicated as “Xa”. BSPH-MHS: Barriers to Seek Professional Help for Mental Health Scale; HSB: help-seeking behavior; K10: Kessler Psychological Distress Scale.

An overview of enrollment, interventions, and assessments is provided in Figure 2.

### Ethical Considerations

The study has been approved by the Institutional Ethics Committee of the National Institute of Mental Health and Neurosciences, Behavioural Science Division (NIMHANS/45th IEC [BEH.SC.DIV.]/2024/22.03.2024) and is prospectively registered in the Clinical Trials Registry of India. Informed consent will be obtained from individuals prior to recruitment, and no compensation will be offered to participants for taking part in the study. Baseline and postintervention surveys will be administered electronically via REDCap (Research Electronic Data Capture), a secure, web-based platform for data collection and management, hosted at the first author’s institution. The digital apps used in the study incorporate “privacy by design” and use technical and policy safeguards to ensure the security of the data and protect the privacy of users. User passwords are stored securely through encryption. Personally identifiable information elements (email addresses and phone numbers) are stored in encrypted form and are decrypted within the program when needed. The data obtained from the participants through text messages and emails will be backed up and stored on a password-protected system.

Only authorized research team members will have access to identifiable data. WhatsApp messages will be standardized and delivered via a business account, with no sensitive information exchanged. Referral-related guidance to seek appropriate professional help will be provided for all individuals who are not eligible for participation and those who decline participation. Participants will receive appropriate mental health service suggestions based on their needs and preferences at any point in time if required. Participants will be free to opt out of the study at any point. A telephone session will be held with those who score above the cutoff on suicidality to guide, motivate, and support them in seeking professional help. They will not be part of the study. Helpline numbers are also available in the app and will be shared with these individuals for use as per their needs. These individuals will also be informed that they can reach out to the researcher again if they need more referral options at a later point. The active control group will be debriefed at the end of the study and provided information and links to the MindNotes app. In addition, they will be free to contact the researcher for guidance or professional help at any point during the intervention period, if they so desire.

### Data Analysis Plan

Quantitative analyses will be conducted using SPSS Statistics (version 31.0; IBM Corp). Baseline characteristics will be summarized descriptively. Differential attrition will be examined by comparing dropout rates between groups using chi-square tests and by assessing baseline differences between completers and noncompleters using appropriate statistical tests. Sensitivity analyses will be conducted as needed based on the observed pattern of missing data.

Occurrence of professional help-seeking at the postintervention assessment and follow-up will be compared between groups using proportions, chi-square tests, and odds ratios with 95% CIs, with Cohen *h* reported as the effect size. As this behavioral outcome has no baseline variation in the nontreatment-seeking study sample, this will be treated as a completer analysis involving participants who provided postintervention assessment data.

For continuous outcomes, linear mixed-effects models will be used to estimate group, time, and group×time effects under an intention-to-treat framework. In addition, completer-only analyses will be conducted as exploratory comparisons to describe observed change among participants with complete data. A 2-sided significance level of .05 will be used.

## Results

The study is part of a project funded by the Indian Council of Medical Research. The funding for the project was received in May 2024. The study was initiated in September 2025. Data collection is expected to be completed by July 2026. The results of the study are expected to be published by December 2026.

## Discussion

### Anticipated Findings

The minimally guided MindNotes app represents an innovative approach to addressing the mental health needs of individuals experiencing psychological distress but who may be uncertain or ambivalent about seeking professional help. This trial seeks to evaluate the app’s effectiveness in enhancing help-seeking, a factor that remains crucial in bridging the treatment gap in low-resource settings such as India [3,15]. In addition, reductions in perceived barriers to help-seeking and psychological distress will be examined because the intervention app includes components directed at learning self-help skills to deal with common mental health concerns, as well as simple tips and strategies to identify and overcome one’s hesitations to seek professional help when needed.

Previous research has highlighted the promise of digital interventions in increasing engagement with mental health care, particularly through the use of behavioral nudges and minimal guidance strategies [14,27]. Building on this evidence, this study tests the combined use of a structured, self-help mobile app with strategically spaced, personalized, and generic reminders. This integrated design is intended to address 2 common challenges in digital mental health interventions: user engagement and retention [33]. The trial is currently in its preliminary phase, with participant recruitment underway. It is anticipated that the study will provide valuable insights into the effectiveness of a minimally guided app-based intervention for hard-to-reach, distressed, nontreatment-seeking individuals in the community [27]. The intervention specifically targets nontreatment-seeking individuals, a group often underserved and unreached by conventional mental health care systems and seeks to evaluate their response to digital support aimed at enhancing mental health awareness, self-help, and help-seeking.

### Strengths and Limitations

The study follows a randomized controlled trial approach, which adds value to the methodological rigor and ensures reliable findings. From an ethical perspective, having an active control group ensures that no participant with distress is denied mental health support. The component of minimal guidance is an advantage of this study as it helps keep participants engaged. Nevertheless, the proposed study has a few inherent limitations. To reach distressed nontreatment seekers, it invites participation from those experiencing psychological distress but not accessing professional help. This approach is likely to appeal to individuals who are ambivalent or in the contemplation stage of seeking help. While the intervention may assist in resolving such ambivalence and encourage movement toward actual help-seeking behavior, those with very limited awareness of their mental health concerns—those in the precontemplation stage—may not be reached. Blinding will not be feasible in this study since the participants will be aware of the nature of the intervention they engage with, and the outcome measures are based on self-reports (eg, help-seeking). It is plausible that while participants’ inclination to seek help may improve, this may not translate into actual professional help-seeking behavior within the 1-month follow-up period. Moreover, help-seeking behavior is influenced by access to affordable professional services (supply-side factors) and other logistical constraints. Although the study will aim to provide appropriate guidance and refer interested participants to relevant local resources, these measures may not always be sufficient to initiate professional help-seeking behavior. Finally, while sustained engagement with mental health services is a key factor in achieving longer-term clinical outcomes, examining the quality of care received or satisfaction with professional consultations falls beyond the scope of this study.

### Future Directions

Limitations of the study notwithstanding, it is expected to make a meaningful contribution to the growing body of literature on mobile mental health interventions in low- and middle-income countries. By leveraging an accessible, scalable, and low-cost indigenous platform, digital interventions such as MindNotes hold promise as viable public mental health strategies in urban Indian community settings, empowering individuals through guided self-help and improving timely help-seeking, particularly when complemented by enhanced access to professional services.

### Conclusions

If proven effective, the study may inform future design, delivery, and policy integration of similar digital tools, helping to address demand-side barriers that contribute to the persistent treatment gap for common mental health concerns in India.

## Abbreviations

BSPH-MHS: Barriers to Seek Professional Help for Mental Health Scale
GHSQ: General Help-Seeking Questionnaire
K10: Kessler Psychological Distress Scale
REDCap: Research Electronic Data Capture
SPIRIT: Standard Protocol Items: Recommendations for Interventional Trials

## Data Availability

The datasets generated or analyzed during this study are not publicly available due to the sensitive nature of the participant data and concerns regarding participant confidentiality and privacy but are available from the corresponding author on reasonable request.
